# CNTNAP3 Associated ATG16L1 Expression and Crohn's Disease

**DOI:** 10.1155/2015/404185

**Published:** 2015-03-26

**Authors:** Yu Qi Qiao, Mei Lan Huang, Qing Zheng, Tian Rong Wang, An Tao Xu, Yuan Cao, Di Zhao, Zhi Hua Ran, Jun Shen

**Affiliations:** ^1^Division of Gastroenterology and Hepatology, Renji Hospital, School of Medicine, Shanghai Jiao Tong University, Shanghai 200127, China; ^2^Shanghai Institute of Digestive Disease, Shanghai 200127, China; ^3^Shanghai Inflammatory Bowel Disease Research Center, Shanghai 200127, China; ^4^Key Laboratory of Gastroenterology & Hepatology, Ministry of Health (Shanghai Jiao Tong University), Shanghai 200127, China

## Abstract

Autophagy is a common physiological process in cell homeostasis and regulation. Autophagy-related gene mutations and autophagy disorders are important in Crohn's disease (CD). The nucleotide oligomerization domain 2–autophagy genes autophagy 16-like 1 (NOD2–ATG16L1) signaling axis disorder contributes to the dysfunction of autophagy. This paper is focused on the relationship between contactin associated protein-like 3 (CNTNAP3) and ATG16L1 expression in Crohn's disease. The results indicated that the expression of ATG16L1 is higher in some CD patients compared to normal controls. ATG16L1 was well correlated with the C-reactive protein (CRP) in some CD patients. In vitro study revealed that CNTNAP3 could upregulate the expression of ATG16L1 and increase autophagy vacuoles.

## 1. Introduction

Autophagy, a common mechanism in cell homeostasis and degradation, has emerged to play a critical role in Crohn's disease (CD). Genome-wide association studies (GWAS) revealed that polymorphisms in autophagy-associated genes such as autophagy-related 16-like 1 (ATG16L1) and immunity-related GTPase family M (IRGM) were risk loci for CD [1, 2]. Autophagy shows the inhibitory role in inflammasome activation, as indicated by the evidence that downregulation of ATG16L1 leads to increased interleukin- (IL-) 1*β* expression in a mouse model of CD [3]. A cohort study suggested that ATG16L1 loci variant displayed increased levels of proinflammatory cytokine IL-1*β* and IL-6 in humans [4]. Another study indicated that pro-IL-1*β* could be specifically sequestered into autophagosomes in macrophages stimulated by toll-like receptor ligands [5]. However, polymorphisms in nucleotide oligomerization domain 2 (NOD2) remain the most prominent genetic risk factor among CD-associated risk loci identified so far [6, 7]. In recent years, several studies have linked the CD-associated NOD2 mutations to autophagy appearance via the interaction of NOD2 with ATG16L1 [8, 9]. NOD2-mediated autophagy is needed in bacterial recognition in the dendritic cells. Besides, both CD-associated NOD2 and ATG16L1 mutative CD patients are defective in autophagy induction, bacterial trafficking, and antigen presentation [8]. During bacterial invasion, NOD2 and ATG16L1 will interact together at bacterial entry sites, initiating the collection of the ATG16-ATG5-ATG12 complex to the autophagosomes. However, mutant CD-associated NOD2 (L1007fsinsC) failed to recruit ATG16L1 to the plasma membrane. Consequently, intracellular bacterial degradation by autophagosomes wrapping is defective [9]. This evidence links NOD2 and autophagy-related genes to innate immune responses against bacterial invasion, showing that the NOD2-ATG16L1 signaling axis disorder contributes to the deficiency of autophagy in CD [10].

Studies have highlighted that autophagy plays the critical role in maintaining intestinal homeostasis, and dysfunction of autophagy seems to be a risk factor in the onset of chronic intestinal inflammation. Some genetic risk polymorphisms have been found related to autophagy. However, most of the autophagy-related polymorphisms were found in specific region or ethnicity. ATG16L1 polymorphism cannot be confirmed as one of the susceptibility loci in CD patients from Asian countries [11, 12]. The selective effects on the cell biology and specialized regulatory properties of ATG16L1 or autophagy in CD patients from Asia are still unclear. Contactin associated protein-like 3 (CNTNAP3) is a gene located in chromosome 9p13.1. Protein encoded by CNTNAP3 gene is a member of NCP family (Neuroxin-IV/CNTNAP/Paranodin) of cell-recognition molecules, a distinct subgroup of the neurexins which mediates neuron-glial interactions [13]. The function of CNTNAP3 has not been fully detected. Due to our preliminary data, CNTNAP3 expression was found upregulated in the intestinal tissue of the patients with CD. However, it is not known how CNTNAP3 contributes to ATG16L1 or autophagy in intestinal biology or CD pathogenesis. Thus, in this paper we try to figure out if CNTNAP3 participates in the process of autophagy or ATG16L1 pathway.

## 2. Materials and Methods

### 2.1. Subjects and Samples

A total of fifteen patients with active CD and fifteen healthy controls (HC) were enrolled in this study from September 2010 to February 2015. The present research was approved by the Research Ethics Committee of Renji Hospital, School of Medicine, Shanghai Jiao Tong University. Written informed consents were obtained from all subjects before recruitment. Patients were diagnosed based on the clinical manifestations, endoscopy, and pathology, confirmed by two gastroenterologists. All patients were newly diagnosed—no medication history. Patients with infectious diseases, pregnancy, or malignancy were excluded. Colonic biopsy specimens were obtained from all subjects. Tissue samples were obtained from inflamed segments of colons in patients and regions without pathological changes in HC. At the same day of colonoscopy, patients provided a fasting blood sample for measurement of serum C-reactive protein (CRP) and erythrocyte sedimentation rate (ESR). CRP and ESR were performed by routine laboratory tests.

### 2.2. Cell Culture and Transfection

HeLa cell line and SW620 cell line were obtained from the American Type Culture Collection (ATCC) and maintained in the Shanghai Institute of Digestive Disease. Both HeLa and SW620 cells were grown in Dulbecco's Modified Eagle Medium (DMEM) (Gibco) with 10% iron-supplemented calf serum (Hyclone), streptomycin (50 *μ*g/mL) (Invitrogen), and penicillin (50 U/mL) (Invitrogen) at 37°C with 5% CO_2_ supplement.

To downregulate the CNTNAP3 expression, small interfering (si)RNA oligos were purchased from GenePharma. The siRNA sequences were designed using GenePharma siRNA design center (http://www.genepharma.com/). These siRNA oligopairs were Cntnap3-homo-2039 (5′-CGUCUGGGCUUUACUAUAUTT-3′ and 5′-AUAUAGUAAAGCCCAGACGTT-3′) and Cntnap3-homo-1311 (5′-GGAAAUGUGUCCUUCUCAUTT-3′ and 5′-AUGAGAAGGACACAUUUCCTT-3′). The negative control pairs were 5′-UUCUCCGAACGUGUCACGUTT-3′ and 5′-ACGUGACACGUUCGGAGAATT-3′. The CNTNAP3 cDNA was amplified and inserted into pcDNA 3.1 Vector (Invitrogen) to establish pcDNA 3.1-CNTNAP3 for overexpression purpose. The plasmids were amplified in One Shot TOP10F' Chemically Competent Cells (Invitrogen) following the manufacturer's guide and purified with PureLink HQ Mini Plasmid Purification Kit (Invitrogen). CNTNAP3 siRNAs or pcDNA 3.1-CNTNAP3 were transfected into HeLa cells, respectively, with Lipofectamine 2000 Transfection Reagent (Invitrogen).

### 2.3. RNA Extraction, Reverse Transcription, and Real-Time PCR

Total RNA from biopsy specimens and cells was extracted using Cell Culture and Tissue Total RNA Extraction and Preparation Mini Kit (SLNco) according to the manufacturer's instruction. The quantity and quality of RNA were confirmed with a NanoDrop 1000 (NanoDrop, Thermo Scientific-Waltham). The primers were designed using Primer Premier 5.0 software and synthesized from Generay Biotech Co., Ltd: CNTNAP3 (forward 5′-TCGCCACCCAAGGAGGATAT-3′, reverse 5′-TCAAAGGGAGGCTGGAGTCTGT-3′); ATG16L1 (forward 5′-AGGACAGGGAGATGCAGATGA-3′, reverse 5′-GATTGGCTTCCTGGGCTTT-3′); *β*-actin (forward 5′-GTCTTCCCCTCCATCGTG-3′, reverse 5′-AGGGTGAGGATGCCTCTCTT-3′). For gene-specific reverse transcription, first strand synthesis was performed with PrimeScript RT reagent Kit (TaKaRa). Quantitative real-time PCRs were conducted on a StepOne Plus device (Applied Biosystems) with SYBR Premix Ex Taq kit (TaKaRa). Data were analyzed by 2-ΔΔCt algorithm [14].

### 2.4. Western Blot

Cell lysates were prepared using RIPA buffer (Sigma) containing protease inhibitors (Roche), subsequently agitated on ice for 30 minutes. Pierce BCA Protein Assay Kit (Pierce) was used to measure the protein concentration. Protein electrophoresis was performed with Mini-PROTEA III (Bio-Rad). In 10% polyacrylamide gels (Tris/glycine), proteins were separated and transferred onto a polyvinylidene fluoride membrane (Bio-Rad). Primary and secondary antibodies were labeled subsequently. Antibodies were applied against CNTNAP3 (rabbit polyclonal anti-CNTNAP3, 1 : 1000, Sigma-Aldrich), ATG16L1 (rabbit polyclonal anti-ATG16L1, 1 : 1000, Abcam), and GAPDH (rabbit polyclonal anti-GAPDH; 1 : 2500, Abcam). Goat anti-rabbit IgG-HRP secondary antibody was purchased from Santa Cruz. Experiments were performed in triplicate.

### 2.5. Autophagy Observation with Fluorescent Microscope

Autophagic vacuoles with monodansylcadaverine (MDC) were stained and assessed as previously described [15]. HeLa and SW620 cells seeded in six- or twelve-well plates were transfected with pSELECT-GFP-LC3 (Invitrogen) using Lipofectamine reagent (Invitrogen) following the manufacturer's instructions. Typically, 1 × 10^6^ cells/mL well in six-well plates was transfected with 0.5 *μ*g plasmids. Transient transfections with cDNAs were performed with Lipofectamine 2000 to label autophagic vacuoles with GFP-LC3 plasmid for 24 to 72 hours [16]. Cells were fixed with 4% paraformaldehyde (PFA) in PBS after the treatments. Images were obtained using a fluorescent microscope (GFP filter-Ex470/40VS495, Em515/30; Zeiss) and GFP-LC3 punctuated dots were determined for triplicates. Quantification of the average number of GFP-LC3 punctuated dots per cell was performed as previously mentioned [17].

### 2.6. Statistics

GraphPad Prism 5.0 for Windows (GraphPad Software) was used in statistics. *P* < 0.05 was considered to be significant with either ANOVA analysis or *t*-test.

## 3. Results

### 3.1. Characteristics of Included Subjects

A total of fifteen CD patients and fifteen healthy controls were enrolled in the present experiment. After we included the CD patients and HCs, we found that CD patients showed a trend of lower body mass index (BMI). The characteristics of included subjects are listed in Table 1.

### 3.2. CNTNAP3 and ATG16L1 mRNA Expression Increase in CD Patients Compared with HCs

**Figure 1:**
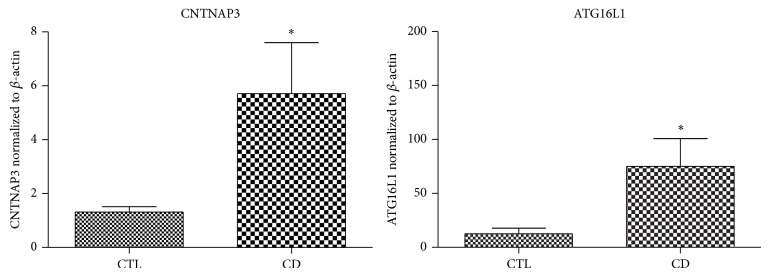
The mRNA expression of CNTNAP3 and ATG16L1 in Crohn's disease. qRT-PCR showed that the levels of both CNTNAP3 (*P* = 0.029) and ATG16L1 (*P* = 0.024) were significantly high in CD patients compared to those in healthy controls.

Expression of CNTNAP3 and ATG16L1 in colonic tissue from CD patients and HCs were detected by qRT-PCR, showing that both CNTNAP3 (*P* = 0.029) and ATG16L1 (*P* = 0.024) were highly expressed in CD patients compared to those in HCs (Figure 1).

### 3.3. ATG16L1 Correlates with C-Reactive Protein (CRP) Levels in CD Patients

**Figure 2:**
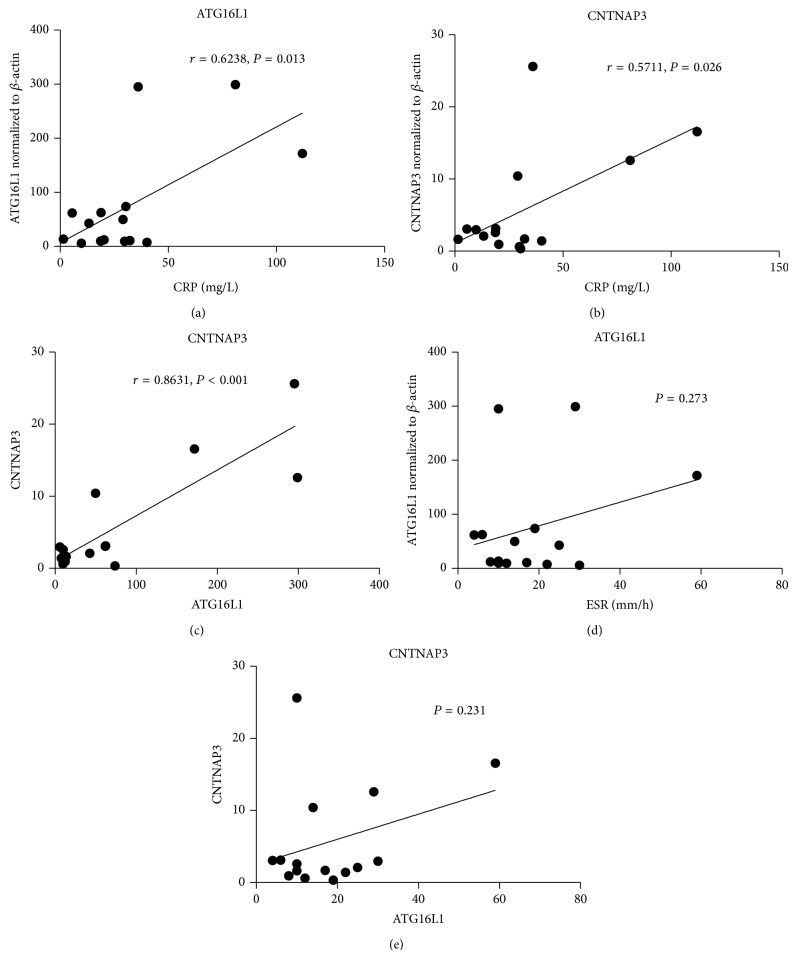
The relationships between CNTNAP3, ATG16L1, and some serum markers. Spearman correlation analysis indicated ATG16L1 mRNA expressions were significantly associated with the serum levels of CRP (*r* = 0.6238 and *P* = 0.013) (a). CNTNAP3 was also showed significance correlated with CRP (*r* = 0.5711 and *P* = 0.026) (b). CNTNAP3 changes positively correlated with the ATG16L1 level (*r* = 0.8631 and *P* < 0.001) (c). Unfortunately, it was found that neither CNTNAP3 (*P* = 0.273) nor ATG16L1 (*P* = 0.231) was significantly correlated with ESR (d, e).

Interestingly, spearman correlation analysis indicated that ATG16L1 and CNTNAP3 mRNA expressions were significantly associated with the serum levels of CRP (*r* = 0.6238 for ATG16L1, *P* = 0.013) (*r* = 0.5711 for CNTNAP3, *P* = 0.026) (Figures 2(a) and 2(b)). CNTNAP3 also significantly expressed correlation with the ATG16L1 transcriptomic level (*r* = 0.8631, *P* < 0.001) (Figure 2(c)). Unfortunately, it was found that neither CNTNAP3 (*P* = 0.273) nor ATG16L1 (*P* = 0.231) was significantly correlated with ESR (Figures 2(d) and 2(e)).

### 3.4. CNTNAP3 Manipulates the Expression of ATG16L1 in HeLa and SW620 Cells

Given the evidence that CNTNAP3 elevated expression was closely associated with ATG16L1, we subsequently changed the expression pattern of CNTNAP3 by knocking down and overexpression, to determine the downstream target, namely, ATG16L1 in HeLa and SW620 cells.

**Figure 3:**
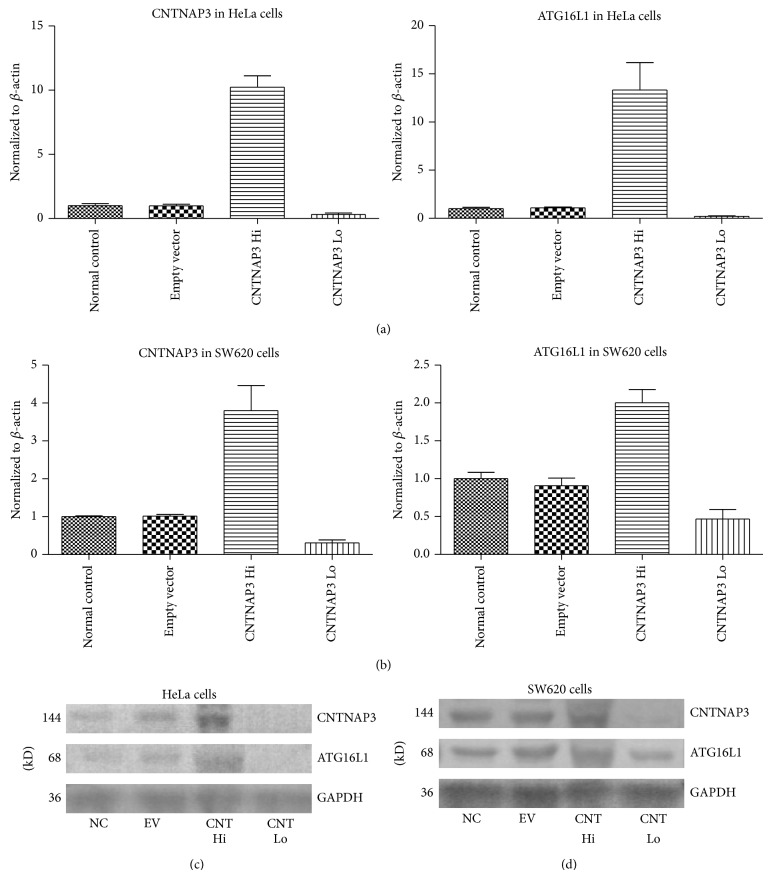
Level of ATG16L1 after CNTNAP3 manipulation. Seventy-two hours after transfection, ATG16L1 was lower in CNTNAP3 knockdown cells than that in cells transfected with empty vectors (HeLa, *P* = 0.013 and SW620, *P* = 0.005). (a and b). On the other hand, ATG16L1 was significantly higher in cells transfected with CNTNAP3 overexpression plasmid than that in cells transfected with empty vectors (HeLa, *P* = 0.001 and SW620, *P* = 0.052) (a and b). There was no significant difference between cells transfected with empty vectors and normal controls (HeLa, *P* = 0.630 and SW620, *P* = 0.517). Similarly, western blot indicated that ATG16L1 protein expression was significantly associated with the expression of CNTNAP3 manipulations. ATG16L1 protein expression significantly decreased and increased 72 hours after CNTNAP3 siRNA interference and CNTNAP3 overexpression, respectively (c and d).

Seventy-two hours after transfection, the level of CNTNAP3 was measured by qRT-PCR (Figures 3(a) and 3(b)) and validated with western blot (Figures 3(c) and 3(d)). Compared with cells transfected with empty vectors, CNTNAP3 was significantly higher in CNTNAP3 overexpression cells (HeLa, *P* = 0.001 and SW620, *P* = 0.014) and lower in CNTNAP3 knockdown cells (HeLa, *P* = 0.013 and SW620, *P* = 0.002). There was no significant difference between cells transfected with empty vectors and normal controls (HeLa, *P* = 1.000 and SW620, *P* = 0.794).

Results showed that ATG16L1 was lower in CNTNAP3 knockdown cells than that in cells transfected with empty vectors (HeLa, *P* = 0.013 and SW620, *P* = 0.005) (Figures 3(a) and 3(b)). On the other hand, ATG16L1 was significantly higher in cells transfected with CNTNAP3 overexpression plasmid than that in cells transfected with empty vectors (HeLa, *P* = 0.001 and SW620, *P* = 0.052) (Figures 3(a) and 3(b)). There was no significant difference between cells transfected with empty vectors and normal controls (HeLa, *P* = 0.630 and SW620, *P* = 0.517).

Similarly, western blot also indicated that ATG16L1 protein expression was significantly associated with the expression of CNTNAP3 manipulations. ATG16L1 protein expression significantly decreased or increased 72 hours after CNTNAP3 was interfered by siRNA and CNTNAP3 overexpressed, respectively (Figures 3(c) and 3(d)).

### 3.5. CNTNAP3 Regulates Autophagy in HeLa and SW620 Cells

To determine whether autophagy can be regulated by CNTNAP3, HeLa and SW620 cells were cotransfected with LC3-GFP plasmid and subsequently observed using fluorescent microscope 72 hours after CNTNAP3 modulation.

**Figure 4:**
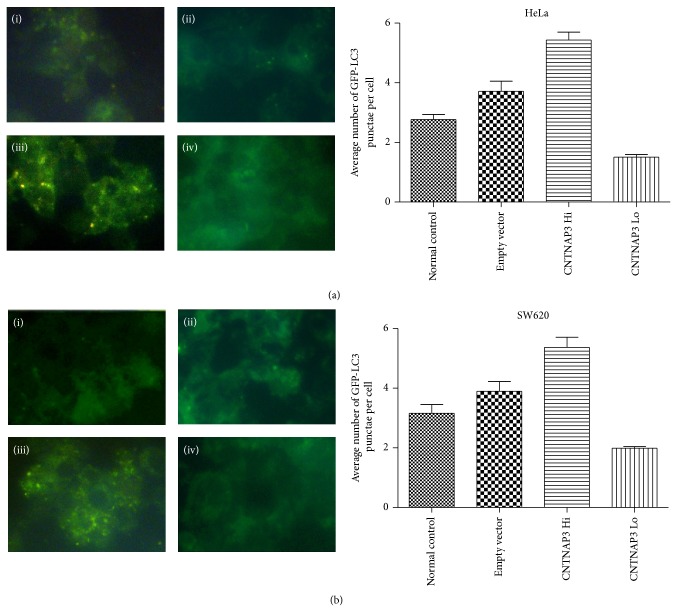
Autophagy phenomenon after CNTNAP3 manipulation. (a) In HeLa cells, the average number of GFP-LC3 punctuated dots per cell of CNTNAP3 overexpression group was 5.434 ± 0.2658, comparing to 1.507 ± 0.08273 in CNTNAP3 knockdown group and 3.714 ± 0.3373 in empty vector group. (b) In SW620 cells, the average number of GFP-LC3 punctuated dots per cell of CNTNAP3 overexpression group was 5.365 ± 0.3412, while it was 1.989 ± 0.05458 in CNTNAP3 knockdown group and 3.899 ± 0.3226 in empty vector group. It was shown that autophagy vacuoles significantly increased (HeLa, *P* = 0.161 and SW620, *P* = 0.036) and decreased (HeLa, *P* = 0.003 and SW620, *P* = 0.004) after CNTNAP3 overexpression and CNTNAP3 siRNA interference compared with cells transfected with empty vectors. ((i) Normal control; (ii) empty vector; (iii) CNTNAP3 overexpression; (iv) CNTNAP3 knockdown).

It was shown that autophagy vacuoles significantly increased (HeLa, *P* = 0.161 and SW620, *P* = 0.036) (Figures 4(a) (iii) and 4(b) (iii)) and decreased (HeLa, *P* = 0.003 and SW620, *P* = 0.004) (Figures 4(a) (iv) and 4(b) (iv)) after CNTNAP3 overexpression and siRNA interference compared with cells transfected with empty vectors (Figures 4(a) (ii) and 4(b) (ii)). No significant difference was found between empty vector group and normal control (HeLa, *P* = 0.066 and SW620, *P* = 0.166). In HeLa cells, average number of GFP-LC3 punctuated dots per cell of CNTNAP3 overexpression group was 5.434 ± 0.2658. It was 1.507 ± 0.08273 in CNTNAP3 knockdown group and 3.714 ± 0.3373 in empty vector group. In SW620 cells, the average number of GFP-LC3 punctuated dots per cell of CNTNAP3 overexpression group was 5.365 ± 0.3412. It was 1.989 ± 0.05458 in CNTNAP3 knockdown group and 3.899 ± 0.3226 in empty vector group (Figures 4(a) and 4(b)).

## 4. Discussion

ATG16L1 is a classic gene that is associated with the development of Crohn's disease. In early research, Hampe et al. identified that ATG16L1 variant was associated with susceptibility to Crohn's disease [1]. And then a nonsynonymous SNP was identified to show association of a threonine-to-alanine substitution (T300A) in ATG16L1. With ATG16L1 (T300A), the innate immune cells cannot be triggered by the specific microbial structures to form the autophagosomes. This phenomenon leads to subsequent bacterial persistence [18]. Interestingly, expression of ATG16L1 was independent of the ATG16L1 (T300A) genotype [1]. Fujita et al. demonstrated that ATG16L1 (T300A) mutant had little impact on canonical autophagy [19]. Although ATG16L1 polymorphisms can lead to subsequent bacterial persistence in the gut mucosa, 25% of Caucasian populations with the risk allele do not have severe gut inflammation [18]. Therefore, ATG16L1 variants could not be the only cause of the disease. On the other hand, many patients with Crohn's disease do not have the risk allele ATG16L1 (T300A). In Italian [20], Brazilian [21], Moroccan [22], Japanese [11], and Korean [12] populations, ATG16L1 (T300A) was not found associated with CD. Although ATG16L1 (T300A) was found in Asian populations, the relationship of the variants and Asian Crohn's disease is still to be confirmed. In some other autoimmune diseases, ATG16L1 was also found upregulated in dendritic cells [23].

In our research, ATG16L1 expression in colonic mucosal samples from CD patients was found to be increased, and mRNA expression of ATG16L1 was correlated with CRP of patients. This phenomenon showed that ATG16L1 played an important role in the course of the disease, and the relationship between ATG16L1 and CNTNAP3 showed that CNTNAP3 might be of importance in autophagy process of Crohn's disease. As newly reported in gut related disease, CNTNAP3 is a distinct subgroup of the neurexins [13]. It was first found in nervous system, the function of CNTNAP3 remaining undiscovered. In our study, we found CNTNAP3 was highly expressed in colonic samples from Crohn's disease patients, and it might increase the autophagy process in cell models. Correlation between CNTNAP3 and ATG16L1 was significantly high. As most reports have confirmed, variants of ATG16L1 gene were related to autophagy deficiency. Contrary to expectation, overexpression of ATG16L1 did not definitely enhance the autophagy. Interestingly, abrupt increase of ATG16L1 could lead to reduced autophagy [24]. ATG16L1 was found to work together with ATG12 and ATG5 in the formation of autophagosomes [25], of which the process was dependent on the correct localization of ATG16L1 to sites of LC3-lipidation. In our research, there were no apparent autophagosomes forming disruption in any group. With the increase of CNTNAP3 and ATG16L1, autophagosomes increased in HeLa and SW620 cells subsequently.

Bacterial immunity was found disabled in autophagy deficiency related to mutations of ATG16L1 or NOD2 [26, 27]. In our research, autophagosomes increased without the stimulation of bacteria. It might be an adaptive reaction of cells under the overexpression of CNTNAP3. Necroptosis of cells might be another explanation. Necroptosis, a programmed cell death which is believed to link with the pathogenesis of IBD, shows the features of necrosis, including mitochondrial swelling and extensive vacuole formation [28]. Autophagy vacuoles are commonly seen during the process of necroptosis.

In our study, biopsies from CD patients showed a higher ATG16L1 mRNA level. ATG16L1 was well correlated with the CRP values in patients indicating that ATG16L1 might be a biomarker for the severity of the disease.

## Figures and Tables

**Table 1:** Characteristic of included CD patients and HCs.

	CD (*n* = 15)	HC (*n* = 15)	*P* value
Age (yrs)	30.27 ± 2.074	29.00 ± 1.604	0.6327
BMI (kg/m^2^)	20.82 ± 0.3448	23.98 ± 0.3927	<0.0001
Gender (female/male)	6/9	8/7	
Smoking	2/13	N/A	
Extent			
Ileocolitis	12	N/A	
Colitis	3	N/A	

CD: Crohn's disease; HC: healthy control; N/A: not applicable.

## Abbreviations

CD: Crohn's disease
NOD2–ATG16L1: Nucleotide oligomerization domain 2–autophagy genes autophagy 16-like 1
CNTNAP3: Contactin associated protein-like 3
CRP: C-reactive protein
GWAS: Genome-wide association studies
ATG16L1: Autophagy-related 16-like 1
IRGM: Immunity-related GTPase family M
NOD2: Nucleotide oligomerization domain 2
Neuroxin-IV/CNTNAP/Paranodin: NCP family
HC: Healthy controls
ESR: Erythrocyte sedimentation rate
ATCC: American Type Culture Collection
DMEM: Dulbecco's Modified Eagle Medium
MDC: Monodansylcadaverine
PFA: Paraformaldehyde
BMI: Body mass index.
